# Editorial: Parkinson's disease: current findings and challenges in diagnosing and treating motor and non-motor symptoms

**DOI:** 10.3389/fnagi.2026.1944463

**Published:** 2026-08-18

**Authors:** Liane Kaufmann, Beatrice Heim, Rejko Krüger, Klaus Seppi, Martin Südmeyer

**Affiliations:** 1 Department of Neurology and Clinical Neuropsychology, Ernst von Bergmann Hospital, Potsdam, Germany; 2 Department of Neurology, Medical University of Innsbruck, Innsbruck, Austria; 3 Luxembourg Institute of Health, Strassen, Luxembourg; 4 Luxembourg Centre for Systems Biomedicine, University of Luxembourg, Esch, Luxembourg; 5 Centre Hospitalier de Luxembourg, Rollingergrund, Luxembourg; 6 Department of Neurology, University Hospital Düsseldorf, Düsseldorf, Germany

**Keywords:** Parkinson's disease, special issue 2026, diagnosis, treatment, trajectories, etiology, translation

Parkinson's disease (PD) is the second-most common neurodegenerative disorder, affecting approximately 2%−3% of individuals aged 65 years or older. Its increasing prevalence, progressive course, and need for long-term multidisciplinary management make PD a major and growing public health challenge. Given the societal impact of PD, it is evident that there is an urgent need for future research efforts aimed at expanding our current knowledge of PD (Poewe et al., 2017).

Rather than representing a single disease entity, PD likely comprises a heterogeneous group of genetic and molecular subtypes that converge on a multisystem neurodegenerative syndrome involving both the central and peripheral nervous systems (Wüllner et al., 2023). Although nigrostriatal dopamine deficiency remains a defining feature, it represents only one component of a considerably broader spectrum of motor and non-motor manifestations. Despite substantial advances in our understanding of PD pathophysiology, accurate diagnosis remains challenging, particularly during early and prodromal disease stages. Likewise, treatment of PD is complex and goes far beyond the cardinal motor symptoms (Schapira et al., 2017; Seppi et al., 2019).

Comprehensive treatment approaches should also target at a wide range of non-motor symptoms, including cognitive impairment, affective disorders such as depression and anxiety, as well as autonomic dysfunctions (Seppi et al., 2019). Current pharmacological and device-aided treatments can provide meaningful symptomatic benefits (Murakami et al., 2023); however, substantial unmet needs persist, particularly for levodopa-resistant axial and late-stage motor symptoms (Heim and Poewe, 2026). Recent advances in drug formulations and delivery systems, novel dopaminergic and non-dopaminergic therapies, pathway-specific treatments, and other candidate disease-modifying strategies are promising but require further clinical validation (Hung and Schwarzschild, 2020).

Upon acknowledging the complex clinical picture and multifaceted nature of PD, it is evident that an integrative perspective that considers risk factors, underlying biological mechanisms, different levels of nervous system involvement, clinical manifestations, disease trajectories, and treatment options is required to advance our understanding of PD (see Figure 1A).

**Figure 1:**
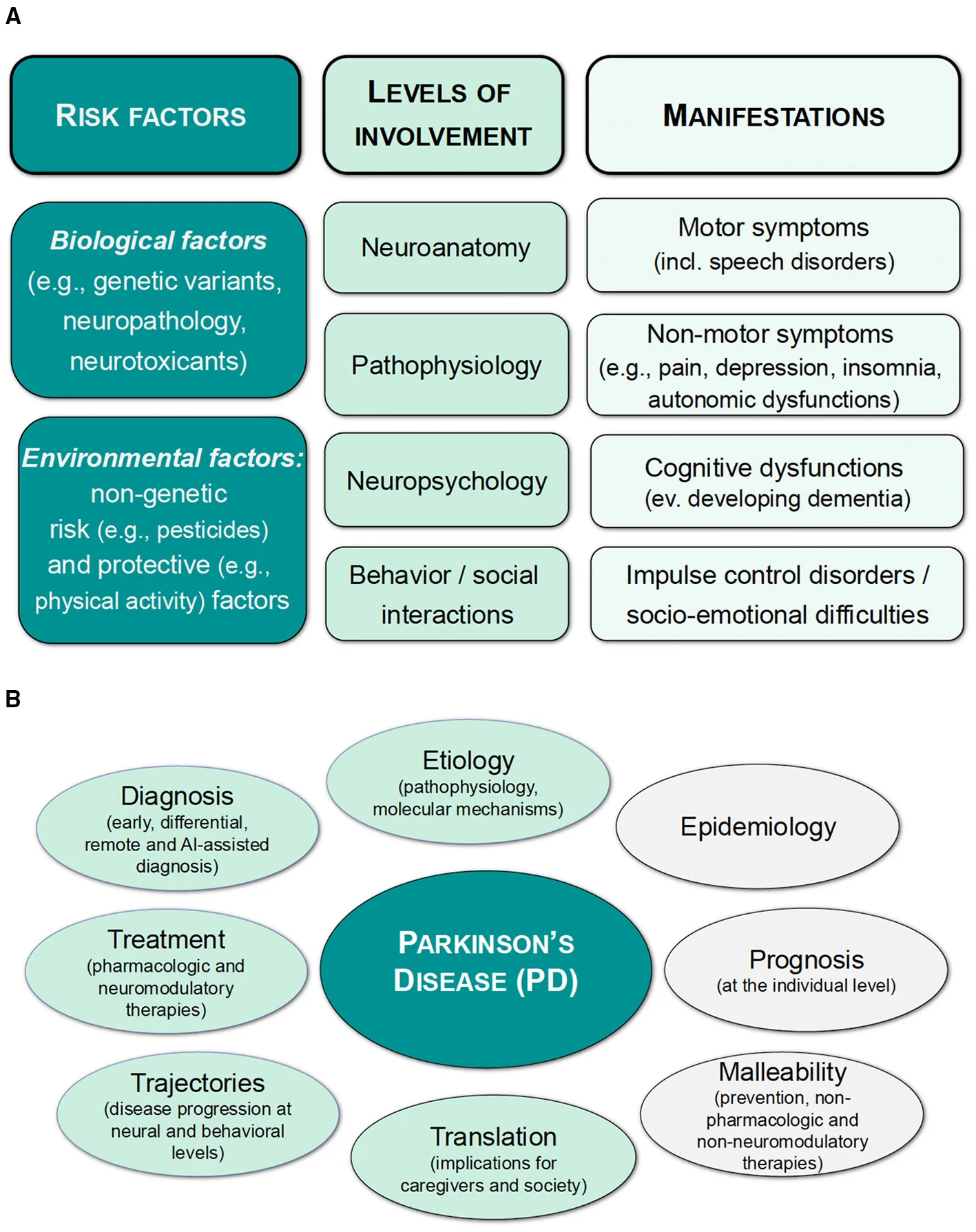
**(A)** An integrative perspective on Parkinson's disease. **(B)** Key areas for future research endeavors targeted at deepening our current understanding of PD. Topics written in light-gray ellipses were not addressed in this Research Topic but are nonetheless relevant and require further systematic investigations.

The present Research Topic was conceived to contribute to this goal. As outlined in the original Research Topic Description, it's principal aims were to “…*enhance our understading of PD by (i) advancing knowledge of neuropathological mechanisms, (ii) elucidating the interplay between motor and non-motor symptoms, (iii) improving early and differential diagnosis, and (iv) elaborating advanced therapeutical approaches.”*

We are pleased to announce that our Research Topic covers a broad spectrum of topics that extends far beyond the aforementioned objectives and also incorporates aspects related to disease progression and translation of research findings into clinical practice (see Figure 1B). The contributions span five major areas of PD research: etiology, diagnosis, treatment, disease trajectories, and translation. At the same time, Figure 1B highlights several important fields that remain underrepresented and warrant further systematic investigation, including epidemiology, individual prognosis as well as non-pharmacologic and non-neuromodulatory interventions. A particular strength of this Research Topic lies in its interdisciplinary nature. It brings together methodologically diverse contributions from neurology, neuroscience, neuropsychology, and the behavioral and biomedical sciences, spanning a broad range of clinical research while also addressing fundamental topics in basic research, including neuroimaging and genetics. In the following sections, we briefly introduce each contribution by assigning them to one of the key areas of PD research depicted in Figure 1B. Because many aspects of PD are closely interconnected, several articles naturally extend across more than one category. Overall, our Research Topic comprises 25 contributions, thereof 15 original research papers, nine review papers (six of which are systematic reviews) and one perspective article.

Contents of this Research Topic (contributions being assigned to the key research topics as depicted in Figure 1B).

## Etiology

Three contributions address issues related to the etiology of PD.

Hanff et al. investigated the modulatory effects of genetic variants (i.e., severe and PD-risk GBA1 carriers) on PD disease progression longitudinally. Findings disclose a trend toward faster cognitive declines in PD-risk and severe GBA1 carriers compared to non-carriers. Moreover, carriers of PD-risk variants displayed a faster decline of tremor and non-motor symptoms than non-carriers and individuals with severe variants.

Uribe-Cano et al. examined the modulating effect of striatal cholinergic interneurons (CIN) on L-Dopa induced dyskinesias (LID) in mice. Their results disclose that—in mice—LID severity and LID-associated molecular markers in CIN may be reduced after D2 ablation from cholinergic neurons. Given that the pharmacological inhibition of D2 generally is associated with debilitating motor side effects, the authors propose that the therapeutic effects of L-DOPA can best be maintained by tuning D2 receptors in CINs at the molecular level. Finally, the authors discuss how their findings could be translated to human studies.

In their systematic review and meta-analysis, Wang et al. aimed to elucidate the association between orthostatic hypotension (OH) and clinical manifestations of PD. Results show that older age, longer disease duration, greater motor symptom severity and poorer cognitive performance are associated with OH in PD. In contrast, no significant differences were found between people with PD (PwP) with and without OH regarding sex, depression, or anxiety.

## Diagnosis

The majority of studies in this Research Topic (*n* = 11) address topics related to the diagnosis of PD, incorporating aspects of motor and/or non-motor symptoms and employing a broad range of methodologies, including self-report questionnaires, neuropsychological assessments, and neuroimaging techniques and digital, remote and AI-based (artificial intelligence) approaches.

The study by Baizabal-Carvallo et al. investigated whether and how self-reported primary motor and non-motor complaints are associated with demographic and clinical characteristics, including cognitive status, among PwP. Findings reveal distinct patterns of self-reported complaints, with cognitive decline emerging as a significant predictor of non-motor symptoms. Although tremor was identified as the most burdensome symptom, nearly 70% of study participants listed one of the non-motor symptoms among their top three complaints.

Xie et al. studied the impact of excessive daytime sleepiness (EDS) on gait disturbances by employing sensor-equipped gait quantification devices. Results show that EDS significantly impairs temporal and spatial gait parameters. Importantly, the association between EDS and gait disturbances is independent from potentially confounding factors. The authors suggest that early detection of EDS could help prevent future deterioration of gait and the risk of falls and therefore conclude that EDS should be part of the routine clinical assessment of PwP.

The study by Zhao et al. also investigated gait analysis, examining the diagnostic utility of quantitative recurrence plots for classifying gait dynamics. Findings demonstrate the high discriminative power of entropy features derived from recurrence plots, with the highest specificity achieved using pressure signals from heel and toe positions. Furthermore, an integrated feature set combining temporal, pressure-based and recurrence plot-based features yielded a maximum accuracy of 92.71% in differentiating PwP from healthy controls.

Sadeghi et al. aimed to identify brain imaging biomarkers for key motor symptoms in PD. Using structural MRI data and quantitative susceptibility maps, the authors conducted multiregional volumetric analyses of two key cortical networks known to be involved in PD motor symptoms (i.e., basal ganglia-thalamo-cortical and cerebellar-thalamo-cortical networks). Findings indicate that network-level structural biomarkers can reliably distinguish PwP from healthy controls and differentiate between tremor-dominant and postural instability/gait disturbance (PIGD)-dominant PD. The authors therefore suggest that brain imaging biomarkers may facilitate early and subtype-specific diagnosis of PD.

Büchel et al. investigated the relationship between decision-making (DM) and other cognitive domains in PD by conducting a systematic review. The results are highly heterogeneous, most likely reflecting the considerable methodological heterogeneity across the studies. Importantly, DM deficiencies need not be associated with deficits in other cognitive domains. Given the broad impact of DM impairments on everyday functioning, including financial and health-related decisions, the authors recommend that DM assessment should be incorporated into routine clinical evaluation of PwP.

In their narrative review, Scanferla and Djamshidian explored impulse control disorders (ICDs) in PwP and how they are modulated by neuropsychological, affective, and behavioral traits. Three main conclusions emerged: (i) ICDs in PwP are not necessarily associated with cognitive dysfunctions; (ii) rather than being a single clinical entity, ICDs are complex manifestations of a heterogeneous spectrum; and (iii) beyond dopaminergic risk factors, other neurochemical systems (e.g., serotonergic markers) appear to play a modulating role in the development and severity of ICDs. The authors conclude that routine, individualized assessment of ICDs may facilitate early detection, risk stratification, and treatment optimization in PD.

Yeshua et al. present a systematic review of social cognition (SC) in PD synthesizing behavioral and neuroimaging findings regarding three key aspects of SC: emotion recognition, empathy/ToM (Theory of Mind), and social problem-solving. Despite considerable methodological heterogeneity and a scarcity of studies targeted at social problem-solving, their findings reveal that already in early PD, emotion recognition and empathy/ToM may be impaired (SC deficiences becoming more pronounced in advanced disease stages). Furthermore, the authors propose a neurofunctional and component-oriented model of SC in PD that could prove useful for future studies.

Using voxel-based morphometry and resting-state network analyses, Danesin et al. examined the cognitive, structural, and neurofunctional correlates of basic and advanced financial abilities (FA) in PwP and mild cognitive impairment (PD-MCI). Their findings revealed distinct cognitive functions and neural networks underlying basic and advanced FA. Although overall performance was high, impairments were more frequent in basic than in advanced FA tasks (18.2% vs. 12.1%, respectively). The authors suggest that this pattern reflects compensatory neural mechanisms supporting advanced FA and propose that financial decision-making in PD-MCI is mediated by adaptive reorganization of cortico-subcortical and cerebellar systems.

Sui et al. investigated cerebral microstructural changes associated with PD non-motor symptoms. To this end, they examined the relationship between depression and anxiety scores in PwP and multiparametric MRI measures, including T1 and T2^*^ mapping, proton density mapping, and quantitative susceptibility mapping (QSM). Findings revealed significant associations between specific quantitative MRI parameters and self-reported depression and anxiety. The authors conclude that multiparametric MRI may facilitate the early detection of non-motor symptoms in PD.

Algon and Saban examined whether remote assessments of cognition and motor symptoms might be used reliably to assess cognitive-motor associations in PD. Findings reveal that home-based assessments (i.e., videoconference-based versions of the MoCA as well as online versions of the UPDRS-III and Hoehn & Yahr scales) provide comparable results to in-person settings by reflecting significant –yet small– negative correlations between cognitive and motor scores. The authors therefore conclude that remote cognitive assessments represent a reliable approach for evaluating disease severity in PwP.

Yang et al. provided an overview of artificial intelligence/AI-assisted assessment tools for the early identification of PD motor symptoms including gait, speech, facial expression, eye movement, handwriting, and finger tapping. Overall, the authors outline the potential benefits of AI-driven technologies for the early diagnosis of PD and ultimately propose approaches for future research aimed at translating experimental, AI-based techniques into clinical practice.

## Treatment

Of five papers addressing the treatment of PD, two empirical studies examine the effects of pharmacological agents, and three review papers analyze the efficacy of neuromodulatory therapies.

Jander et al. assessed the applicability of subcutaneous foslevodopa/foscarbidopa (LDp/CDp) therapy—a standard treatment for advanced PD—in a day-clinic setting. Results of this retrospective study reveal that, compared with optimized oral treatment, LDp/CDp provides significantly greater improvements in motor symptoms, leading to enhanced mobility and activities of daily living. The authors therefore conclude that LDp/CDp can be effectively implemented in a day-clinic setting, provided treatment regimens are tailored to the needs of PwP and their caregivers.

Oehlwein et al. explored the long-term outcomes of an amantadine-based combination therapy by collecting clinical data of PwP over a period of up to 13 years. Findings reveal initial and persisting benefical effects on motor symptoms and reduction of most adverse effects. The authors conclude that future research should directly compare the efficacy and safety of amantadine-based combination therapy with standard levodopa therapy in PwP.

Using a systematic review, Jost et al. examined whether transcutaneous auricular vagus nerve stimulation (taVNS) can improve cognition in individuals with neuropsychiatric disorders. The included studies comprised individuals diagnosed with PD, MCI, Major Depression, COVID-19, long-COVID, and Epilepsy. Overall, taVNS was associated with improvements across multiple cognitive domains, including global cognition, attention, memory, language, executive function, and social cognition. However, the authors caution that these findings should be interpreted carefully because of substantial methodological heterogeneity across the included studies.

In their systematic review and meta-analysis, Ye et al. evaluated the effects of transcranial alternating current stimulation (tACS) on neurophysiologic motor function in PD. Their findings indicate that tACS has beneficial effects on motor functions, as reflected by improvements in motor evoked potentials and short-interval intracortical inhibition. The authors suggest that tACS is an effective treatment method for alleviating motor symptoms in PD and should therefore be integrated into PD rehabilitation.

Hofacker et al. conducted a narrative review to investigate whether deep brain stimulation (DBS)—an established surgical intervention to improve PD motor symptoms—may alleviate dysphagia in PwP. Dysphagia is a common complication in PD that seriously hampers medication intake. Findings are heterogeneous and disclose that the beneficial effects of DBS on dysphagia depend on various factors, including the stimulation site (subthalamic vs. pallidal DBS). Interestingly, subjective reports of swallowing improvement following DBS were more positive than objective measures of swallowing function.

## Trajectories

Two papers aimed at investigating disease trajectories in PD by focusing on cognitive functions.

Gibbs et al. examined the longitudinal relationship between cognition and non-tremor motor symptoms in PD, using seven assessments conducted at 3-month intervals. Findings reveal that though MoCA scores were not significantly correlated with severity of motor impairment, global cognition at baseline was associated with progression (but not severity) of PD motor symptoms. However, interestingly, the latter link was observed only for non-tremor motor symptoms. The authors interpret their findings as evidencing partly distinguishable neurofunctional circuits underlying non-tremor and tremor motor symptoms in PD.

Peña Arauzo et al. conducted a systematic review to investigate whether cognitive deterioration may precede the diagnosis of PD. The results indicate that cognitive impairments can occur during the prodromal phase of PD, with these being most consistently observed in tests tapping executive functions, processing speed and attention. However, the authors emphasize that large-scale, long-term follow-up studies are needed to further clarify whether—and which—cognitive domains are most susceptible to be hampered in prodromal PD.

## Translation

Four studies address the translation of research findings into practice; three dealing with aspects of care for PwP and one examining the use of digital health applications among PwP.

Fründt et al. explore long-term nursing care (LTC) for PwP in Germany. Beyond examining the quantity and quality of LTC for PD, the authors assessed the nursing staff's knowledge on PD. The findings provide a comprehensive overview of current care practices, including prior experience with PD, the number of PwP receiving care, the average daily care time per individual, as well as perceived challenges such as chronic staff shortages, high staff turnover, and insufficient PD-specific knowledge. The authors conclude by discussing strategies to improve LTC for PwP.

In this perspective, Ghilardi et al. describe the current state of care for Hispanic PwP in the US and identify factors contributing to gaps in care and low research participation. Recognizing the importance of community-based organizations, the authors propose a model that offers strategies to promote better care and increased research participation among Hispanic PwP in the US.

Wellach et al. examined outpatient care practices for PwP in Germany. The results of their online survey reveal, among other things, an imbalance in the diagnosis and treatment routines for PD motor and non-motor symptoms, with the latter receiving insufficient attention. The authors therefore emphasize the need to further refine outpatient care guidelines to improve treatment outcomes.

Giraitis et al. examined the engagement of PwP with Luxembourg's national electronic health record (EHR) system. The EHR is designed to facilitate information exchange and personalized medical decision-making. Their survey findings indicate that use of EHRs among PwP depends on multiple factors, greater use being associated, among other things, with lower disease severity, higher digital health literacy and support from health-care-providers in using the EHR. The authors therefore conclude that promoting EHR adoption requires addressing individual-, healthcare provider-, and technology-related factors.

Overall, this Research Topic provides a broad and timely overview of contemporary PD research, reflecting both the diversity of current scientific approaches and the increasing integration of basic, translational, and clinical perspectives. While the collected contributions showcase important advances across multiple domains, they also identify key areas that deserve greater attention in future research. We therefore hope that this Research Topic will not only serve as a valuable resource for researchers and clinicians but also stimulate interdisciplinary collaborations that accelerate the translation of scientific discoveries into meaningful improvements in the diagnosis, treatment, and long-term care of PwP.
